# Causal models in epidemiology: past inheritance and genetic future

**DOI:** 10.1186/1476-069X-5-21

**Published:** 2006-07-21

**Authors:** Paolo Vineis, David Kriebel

**Affiliations:** 1 Division of Epidemiology, Public Health and Primary Care, Imperial College London, Faculty of Medicine, Imperial College London, St Mary's Campus, Norfolk Place, London, W2 1PG, UK; 2 Department of Work Environment, School of Health and Environment, University of Massachusetts Lowell, Kitson Hall, Room 200 (UML North), 1 University Avenue, Lowell, MA 01854, USA

## Abstract

The eruption of genetic research presents a tremendous opportunity to epidemiologists to improve our ability to identify causes of ill health. Epidemiologists have enthusiastically embraced the new tools of genomics and proteomics to investigate gene-environment interactions. We argue that neither the full import nor limitations of such studies can be appreciated without clarifying underlying theoretical models of interaction, etiologic fraction, and the fundamental concept of causality. We therefore explore different models of causality in the epidemiology of disease arising out of genes, environments, and the interplay between environments and genes. We begin from Rothman's "pie" model of necessary and sufficient causes, and then discuss newer approaches, which provide additional insights into multifactorial causal processes. These include directed acyclic graphs and structural equation models. Caution is urged in the application of two essential and closely related concepts found in many studies: interaction (effect modification) and the etiologic or attributable fraction. We review these concepts and present four important limitations.

1. Interaction is a fundamental characteristic of any causal process involving a series of probabilistic steps, and not a second-order phenomenon identified after first accounting for "main effects".

2. Standard methods of assessing interaction do not adequately consider the life course, and the temporal dynamics through which an individual's sufficient cause is completed. Different individuals may be at different stages of development along the path to disease, but this is not usually measurable. Thus, for example, acquired susceptibility in children can be an important source of variation.

3. A distinction must be made between individual-based and population-level models. Most epidemiologic discussions of causality fail to make this distinction.

4. At the population level, there is additional uncertainty in quantifying interaction and assigning etiologic fractions to different necessary causes because of ignorance about the components of the sufficient cause.

## Background

In setting health policy priorities, it is crucial to know how large a burden of disease should be attributed to each particular preventable cause. For example, the occupational burden of cancer has been the subject of detailed studies [1,2]. Unfortunately, there are substantial uncertainties in nearly all the data that are used in making these estimates. The key data components are: 1. – a list of all of the known or suspected carcinogens in the workplace; 2. – the prevalence of exposures to these agents; and 3. – information on the magnitudes of the risks of different types of disease from the various exposures (exposure response curves). Major gaps exist in what we know about all three of these, and so considerable humility is called for when assigning a figure to the occupational cancer burden and other types of environmental diseases. Additional uncertainties of a more fundamental nature also play a role: how to factor into such estimates the possibility (or indeed strong likelihood) that multiple risk factors interact in ways that we do not understand, thereby preventing a simple summing of the etiological contributions of different causes. The challenge is not limited to cancer studies: the problem of identifying and quantifying multiple component causes of disease is one of the most basic limitations in modern epidemiology.

### Concepts of "causality" in medicine: from Koch to Rothman

In a rather simplified way, causation involves the relationship between at least two entities, an agent and a disease. Historically, at least two distinct eras of medical causality can be distinguished. The first era corresponds to the 'microbiological' revolution (i.e., the triumph of a linear monocausal (Aristotelian) concept of cause). After the work of Pasteur and Koch, the agent of a disease came to be conceived of as a single necessary cause (e.g. Mycobacterium for tuberculosis). The concept of necessary cause means that the disease does not develop in the absence of exposure to the agent. Such a view implies: a) that the cause is, at least potentially, definable unequivocally and is easily identifiable, and b) that the disease can be also defined unequivocally (i.e., it is not a complex and variable constellation of symptoms). Clearly there are some conditions in which the relationship between a (necessary) cause and the corresponding disease is indeed evident: for example, smallpox is a clear-cut disease entity, easy to define and diagnose, due to a single necessary virus (no smallpox develops in the absence of the specific virus) and clear proof of the causal link has come from the disappearance of smallpox after large scale vaccination.

Cases such as smallpox are, however, an exception. More frequently, in the "Pasteur-Koch" paradigm, we find a clearly defined agent (usually a bacterium, parasite or virus), which is used as the "unifying element" of a *constellation *of symptoms (i.e., the disease, say, streptococcal throat infection, itself is largely defined and recognized on the basis of the agent). Although, the popularity of the "Pasteur-Koch" approach to causality has not decreased, and the concept of a necessary cause of disease is still discussed as a universal paradigm in medicine.

The second era in the history of causation in medicine arises out of the study of chronic diseases like cancer or cardiovascular disease. In these cases, the concept of a "necessary" condition is rarely, if ever, meaningful. No "necessary" cause of cancer is known (with the possible exception of human papilloma virus and cervical cancer); rather, in such cases, the idea of a "causal web" has been introduced and widely applied [3]. The causal web reflects the fact that a concurrence of different "exposures" or conditions is required to induce disease, none of which is in itself necessary. For example, lung cancer can be induced by a causal web, including tobacco smoking and individual predisposition from CYP1A1 and other high-risk genotypes [4]. Another causal web may be represented by asbestos exposure and low consumption of raw fruits and vegetables in the occurrence of mesothelioma. The idea of the web implies that while the disease is usually well-defined from a clinical point of view (e.g. lung cancer or mesothelioma), the etiologic perspective is more complex: not all lung cancer cases can be linked to the same exposures, but may instead share partially overlapping constellations of causes.

The main causal model used by epidemiologists today is Rothman's "pies" [5]. The idea is that a sufficient causal complex (a pie) is represented by the combination of several component causes (slices of the pie). A set of component causes occurring together may complete the "pie", creating a sufficient cause and thus initiating the disease process. Rothman's model has been useful on several accounts. For example, suppose three factors (A, B and C) make a sufficient cause of disease X. Then, one can see that A will appear to be a stronger or weaker cause depending on how common the other "slices" B and C are. A will have a large impact on disease occurrence in a population in which B and C are common, but no effect at all (though being a sufficient cause), where B or C is absent. If it were true that the sufficient cause A+B+C were the *only *pathway to disease X, then it would follow that blocking or eliminating any of these three factors would prevent the disease. Thus, A and B and C would be *necessary *component causes. But if A, for example, also contributed to a sufficient cause with factors D, E and F, then blocking B would not prevent disease X. This more complex view (many pies to which factors contribute) is supported by the epidemiologic evidence for most chronic diseases. There are only few examples of necessary component causes for cancer or heart disease.

The above considerations concern our understanding of disease causality at the *individual level*. The model looks different if we shift from the individual to the *population*. Here, the idea of single "necessary" components makes sense. If we consider the current epidemic of lung cancer, for example, there is no doubt that it is attributable to the diffusion of the habit of tobacco smoking. For, although we cannot attribute any single case of lung cancer to that individual's smoking habits, there is no doubt that, on a population level, the epidemic would not have occurred without cigarette smoking. Notice that this assertion is not contradicted by the fact that lung cancer also occurs among non-smokers. Indeed, the evidence for cigarette smoking as a (population level) cause of lung cancer is quite strong: the risk of cancer in those who stop smoking decreases considerably, in comparison with continuing smokers, and, after a few years, approaches the risk of non-smokers [6]. It should be clear then, that we have to apply different criteria of causation when considering the causes of disease at the individual or population level. We can say that for chronic diseases, the model of causal complexes in which there are necessary components is valid at the population level.

Another difficulty with Rothman's pies is that they do not tackle the temporal sequence at all. We therefore have to consider other models.

### Intermediate variables

At least three modern causality models deserve consideration: graphical models (as e.g. in Pearl's approach) [7], counterfactual models [8] and structural equation models [9]. These models are worth mentioning, because they have added some layers of complexity to the discussion on causality and have also contributed to solving some outstanding issues, including a more sophisticated approach to confounding and identification of intermediate variables.

One of the main challenges to correctly identifying causal sequences involving potential intermediate variables (like biomarkers) is to assess whether the "intermediate" variable belongs to the causal pathway between exposure and disease, or whether it lies on a separate pathway, correlated in some way with exposure or disease. Explanations for the biomarker's association can include confounding, since the epidemiologist's view of the process is always a population perspective. For example, certain mutations may constitute genuine intermediate markers in causal pathways between certain chemicals and cancers, whereas other mutations are a consequence of a different chain of events, like genomic instability that arises in cancer cells (i.e., an effect of the disease, not a cause). As an example of probable confounding, it has been shown that C-Reactive Protein (CRP) levels change with changing levels of other markers of inflammation and with levels of exposure to environmental risk factors for heart disease [10]. It is not clear whether CRP itself lies within the causal pathway or is only a confounded marker for other changes. The distinction is of critical importance in epidemiology: if the biomarker is a confounder, then its effect should be controlled to produce less biased estimates of associations in the causal web. If, however, the biomarker is on the causal pathway, then controlling for it will *introduce *bias, of potentially substantial magnitude.

Very often intermediate events are both causal events *and *confounders, thereby complicating resolution of causality webs. For example, the development of respiratory disease (measured for example by a change in forced expiratory volume in the first second (FEV_1_)) is an independent predictor of both mortality and subsequent weight loss, and is influenced by prior weight gain [11].

One statistical approach to disentangling confounding uses structural equation models based on the logic of counterfactuals [8]. The basic idea is that the exposure leading to changes in the intermediate marker could be theoretically randomized to create the counterfactual instance, in which those with the marker and those without have exactly the same levels of exposure. This approach would enable us to distinguish a genuine intermediate marker (e.g. CRP) from one that is confounded by exposure. Typically, this solution is only a "thought experiment," because most exposures cannot be randomized, except for certain preventive or therapeutic interventions. In the absence of real randomization, the approach involves creating a system of equations which, under certain assumptions, can estimate the counterfactual set of outcomes that each subject would have experienced if (s)he had experienced exposures other than the one actually received.

**Figure 1:**
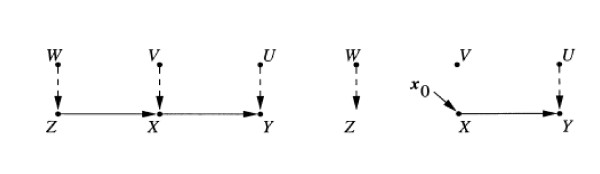
**An example of DAG**. From reference 7. The letters indicate "nodes" in the graph and stand for variables in the causal model. Arrows ("edges") represent relationships. Unobserved exogenous variables are connected by dashed arrows.

A similar approach founded on counterfactuals uses graphical methods [7]. According to Pearl [7], a causal graph "is a directed acyclic graph (DAG) in which the vertices (nodes) of the graph represent variables and the directed edges (arrows) represent direct causal effects" (Figure 1 is an example). The main objective of DAGs is to separate the language of statistical association from the language of causality, by making the latter explicit in a graphical form. As expressed by Pearl [7], the statistical language does not permit us to distinguish between statistical dependence, quantified by conditional probabilities, from causal dependence, "for which we have no expression in standard probability calculus". The first to apply this approach was the geneticist, Sewall Wright, who noticed that equations are symmetrical objects (i.e., they can be rewritten in order to exchange the dependent and the independent variables); therefore, Wright complemented equations with a "path diagram" [7].

The notation of DAGs has the merit of making causal assumptions explicit. Through the separation of different pathways in the graphical structure, it is possible to simulate an experiment (even in the absence of randomization) and, thus, separate causality from confounding. The effect is defined as "the capacity to transmit changes among variables." As Figure 1 shows, if we separate Z from X, we can evaluate whether X is a genuine causal factor or if it is confounded by Z. The joint distribution associated with the modified model describes the post-intervention distribution of variables (i.e., the controlled or experimental distribution): if X represents a treatment variable, Y a response variable, and Z some covariate that affects the amount of treatment received, the post-intervention distribution assesses treatment efficacy by comparing aspects of the distribution at different levels of X.

Once the paths are clarified and separated, one can write a series of equations which describe them, and use these equations to estimate the relevant associations. These equations will be valid under two assumptions: (a) the graph is acyclic and (b) all the error terms are jointly independent. Unfortunately, both assumptions are frequently violated in epidemiological research: (a) very often feed-back, (for example, circularity) is encountered (e.g. in the example of obesity, which causes cardiovascular disease, which in turn leads to weight loss), and (b) errors are not independent. But when conditions (a) and (b) are met, we can predict post-intervention distributions from pre-intervention distributions, even in the absence of real intervention (i.e., of a randomized trial).

### Interactions

"Interactions" have frequently appeared in the literature of chronic disease epidemiology, but many of these papers address statistical issues, such as the type (additive or multiplicative) of the joint effect of variables, providing very little insight into the underlying (biologic) mechanisms that could justify the choice of a model. Pathophysiologic mechanisms of important chronic diseases are usually complex and, for the most part, poorly understood, but one general principle seems to apply: the interaction among the component causes occurs dynamically over time. The development of disease involves an essential temporal sequence of initiation and subsequent stages, and this fact has rarely been appreciated in epidemiologic considerations of interaction [12]. Exposure effects may be very different, when the exposure acts upon a population whose members are not at the same stages along a causal pathway, and those stages may not be known to the researcher. The outcome will appear as heterogeneity (potentially quite severe) in susceptibility, when viewed statically at a single point in time as when epidemiologists determine the momentary incidence and compare the exposure histories of study subjects.

Accordingly, there are two fundamental types of methodological inadequacy in current epidemiologic investigations of interaction. One derives from simple ignorance of the underlying biological processes. The second, typically addressed by epidemiologists, has to do with measurement problems: imprecision of variables, misclassification, and the lack of power of most studies focused on the investigation of interaction [13-15]. We believe that the first is likely to be much more important, although it has received less attention. This issue may be best illustrated by gene-environment interactions.

#### A taxonomy of gene-environment interactions

Quite possibly, genetic component causes play a role of any disease, even those (like lung cancer) that also have important environmental causes. As an example of the opposite situation, homozygotes for the phenylketonuria (PKU) mutation have a deficiency in the enzyme required to convert phenylalanine to tyrosine. If untreated, they will accumulate phenylalanine in the blood and develop mental retardation, but careful dietary restriction can keep phenylalanine concentrations low and thereby prevent retardation. Thus, it is particularly important to understand how genetic and environmental factors may contribute to the same sufficient causes, or more generally, how they interact.

**Figure 2:**
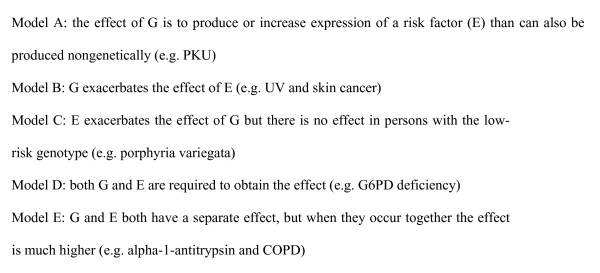
Ottman's taxonomy of gene-environment interactions (G = genotype, E = environment).

An effective taxonomy to describe gene-environment interactions (irrespective of their being additive or multiplicative) has been provided by Ottman [16] (Figure 2). Based on concrete biologic knowledge, the genotype G in Ottman's Model A produces or increases the expression of a risk factor or effect (E) that can also be produced or prevented) nongenetically, as in the case of PKU. In Model B, G exacerbates the outcome of E. For example, xeroderma pigmentosum is an autosomal recessive disorder in which exposure to ultraviolet (UV) light causes a high incidence of skin cancers, due to a defect of DNA repair. However, skin cancer is also associated with UV exposure in people without this disease. In Model C, E exacerbates the effect of G, but there is no effect in persons with the low-risk genotype. For example, an autosomal dominant disorder, porphyria variegata, is characterized by severe skin problems. Exposure to barbiturates strongly exacerbates the symptoms and can lead to death. In Model D, both G and E are required to obtain the effect. Deficiency of glucose-6-phosphate dehydrogenase is an X-linked recessive disorder: individuals are asymptomatic, unless they eat fava beans, in which case they develop severe hemolytic anemia. Fava beans do not produce any symptoms in normal individuals. Finally in Model E, G and E both have separate effects, but when they occur together, the outcome is more severe. For example, the risk of chronic obstructive lung disease is increased in smokers without alpha-1-antitrypsin deficiency and in non-smokers with the deficiency, but risk is greatly increased in smokers with this enzyme deficiency. We suspect that most gene-environment interactions relevant to environmental exposures and common chronic diseases belong to category E.

#### Limitations of statistical models

In the epidemiology of chronic disease, different external exposures may show their effects in an additive or a multiplicative manner (i.e., the joint effect of two or more exposures, or of a genetic factor and an environmental one is the sum of their separate effects or their product, or something else) [13]. Resolution of this issue has been difficult, partly because of a lack of high quality data from sufficiently large studies, and partly because of the difficulty in identifying underlying biologic phenomena from statistical models. Thus, the pathogenesis as the biologic aspect (i.e., how different component causes combine) occurs within individuals, while the statistical models evaluate population data. There is still much to learn about how to make inferences across these two levels of observation.

The biostatistical models for evaluating interaction – usually multivariate regression models – are based on the analysis of variance. This most obvious case is the ordinary least squares multiple regression model, while the same is true as well for the epidemiologist's preferred tools – the logistic, Poisson, and Cox regression models. These all estimate parameters by quantifying (or partitioning) the amount of the variation in risk that should be attributed to one or another independent covariate. In structure, these models are generally linear – thus, implicitly assuming that the "main effects" of two or more environmental exposures, or of several genetic and environmental factors, will combine additively in affecting disease. Variances are computed, and the role of the two main effects (or their interaction) is apportioned accordingly.

Lewontin argues that the analysis of the variance approach is often misleading [17]. There is no theoretical justification for the presumption of a linear explanation (this is done for the sake of simplicity, but is not generally based on any formal biologic assumptions). In an essay entitled "The Analysis of Variance and the Analysis of Causes", Lewontin cites experimental data to argue that mutations often cause a change in what is called the "norm of reaction", or the ability of the organism to react to different environmental conditions. The way in which the mutant strain will react, say, to different temperatures, is not consistent or predictable across the range of varying environmental conditions. A non-linear model may therefore be needed to describe the interaction between a change in genotype and a change in environmental conditions. Thus, analysis of variance will correctly correspond to an "analysis of causes" (i.e., quantifying the relative importance of the main effects of genes, environment and their interactions) only when: (a) environmental exposure-response relationships are linear for individuals with each of the different genetic polymorphisms, and (b) the study includes a sufficiently broad range of exposures to provide statistical power to detect an interaction.

Considering Ottman's five models of interactions, the first of these two conditions will only hold for models D and E. Thus, in the absence of detailed knowledge of biological mechanisms of disease and the roles of environmental exposures and gene polymorphisms, it will generally be inadvisable to use standard statistical models to apportion variance in evaluating gene-environment interactions.

### Attributable fractions

Predictions of the amount of disease that would be prevented if a certain factor were blocked or eliminated from the causal web are often called attributable fractions [18]. Greenland and Robins (1988) have shown that the quantity that is typically estimated by epidemiologists (which they call the excess fraction) is different from (and in most cases less than) the etiologic fraction or that proportion of the disease burden that is causally related to the exposure [19]. The standard methods will generally not estimate the proportion of cases which are etiologically related to an exposure; generally, they will underestimate this quantity by an unknown amount. There are several limitations of the standard methods, but the most important is that the usual formulas cannot account for the possibility that an exposure may move forward in time the onset of a case that would have occurred eventually in the absence of exposure.

As an appropriate example, suppose we are interested in estimating the contribution of a workplace allergen to the rate of asthma in an occupationally exposed cohort. Without strong biologic assumptions, it is not possible to say whether there were new cases of asthma in the study period that would have occurred in the absence of the exposure, but whose time of onset was advanced by the exposure. To include this kind of "etiologic case" (as Greenland and Robins call it) in the total burden of the exposure seems appropriate, although that would require strong biologic assumptions. Again in this case, the interpretation of attributable fractions must be cautious given the limited understanding that epidemiologists (and science in general) have of how to study the temporal dynamics of causal processes.

### Main effects

Epidemiologists tend to focus primarily on the "main effects" of single exposures, when analyzing the role of the environment in causing disease [20]. By "main effect," we mean the fact that the contrast of interest is between those exposed to a single environmental agent and those unexposed, irrespective of other exposures or genetic variations. Interactions are considered to be something secondary, if not an interference, to the direct and (unicausal) association of interest.

The "main effects first" strategy may appear parsimonious, but it is inconsistent with what we know about the mechanisms of carcinogenesis and other chronic diseases, and also with common-sense reasoning about causality. In carcinogenesis, for example, it is well-established that the pathway to a tumor includes several stages, and that some exposures can lead to cancer by "completing" the causal chain already initiated by previous exposures. This implies a lack of independence between "earlier" and "later" causes that would seem to conflict with an approach which views interactions as of secondary importance. In general, we are probably constantly affected by "incomplete" causal chains, which can be precipitated by timely causal events.

#### Mr. Smith's house on fire

Here is a common-sense example, which may help to illustrate why interactions cannot be secondary phenomena in any causal process consisting of a chain of steps. Let us suppose that Mr Smith is quite absent-minded, so he often leaves the gas oven in his kitchen on. Let us also suppose that his house is equipped with a fire alarm and, if this is working, let us say for the sake of simplicity that fire fighters will always arrive and extinguish any fire. If, however, the gas is alight and the alarm is not functioning, the probability of a fire is 1 (100%).

There are several scenarios that we can imagine. To start, we establish some *a priori *probabilities of certain events occurring in an interval of time (for example. one day):

A. the probability that Mr Smith leaves the gas alight is 50%, or p(A) = 0.5

B. the probability that the alarm system does not work is 1%, or p(B) = 0.01

C. the probability that a fire develops for reasons other than those considered here (the "background risk") is 1/1,000, or p(not A and not B) = p(C) = 0.001

With these assumptions, we can easily calculate the risk of fire under various scenarios:

#### 1. The scenario of ignorance

If Mr Smith does not remember whether he left the gas on, and he does not know if the alarm works, then the probability of a fire occurring through the causal chain involving these two factors is:

p(A and B) - p(not A and not B) = (0.5 × 0.01)-0.001 = 0.005-0.001 = 0.004.

This figure is analogous to an attributable risk, as it expresses the probability of the event occurring through some specific mechanism or causal chain. The *relative *risk of a fire occurring through this chain, compared to the risk of fire through some other causal chain (C, the "background risk") is 0.005/0.001 = 5.

#### 2. A scenario of partial knowledge

If Mr Smith knows that he left the gas on, but he does not know if the alarm works, then the probability of a fire is:

p(B given A) - p(non-A and non-B) = 0.01 - 0.001 = 0.009.

The relative risk for this causal chain compared to the background risk is 0.01/0.001 = 10.

#### 3. The scenario of perfect knowledge

If Mr Smith knows both that he left the gas on AND that the alarm does not work, then the probability of a fire is 1, the probability that the fire arises as a consequence of this particular causal chain is 1 - 0.001, and the relative risk is 1/0.001 = 1000.

In all of these simple calculations, we assume the independence of A and B (i.e., absent-mindedness has nothing to do with malfunctioning of the alarm). Although this example is overly simplistic, it is relevant to the problem of attributing cancers to particular causes. We know that cancer requires several stages to develop, and we can imagine that some of the exposures that lead to cancer are common (around 50%, like cigarette smoke) and others rare (like some genetic traits). However, what really counts is their combination, and in particular the fact that some exposures can "complete an incomplete causal chain". What makes this insight particularly important for the problem of attributing causes of a disease is that while we are confident that multiple factors act through causal chains such as these, we are almost always quite ignorant about what components make up these chains, whether they must act in a particular temporal order, and so on.

Returning to the house fire example, notice the impact of knowledge on the relative risk. In the scenario of ignorance, in which we did not know whether the gas had been left on nor whether the alarm would function, we obtained a relative risk of 5 by comparing this causal chain to the background risk. If we had partial knowledge – for example, we knew that the gas was on, but not whether the alarm would function – the relative risk increased to 10. Finally, if we also knew that the alarm was broken, then we could be certain that a fire would develop.

The relevance of these arguments to the previous discussion is that they suggest that interaction is not a secondary property that can be expressed only according to some mathematical (additive or multiplicative) model, but it is simply the ability to complete an incomplete causal chain. The fact that we assumed that absent-mindedness had nothing to do with whether the alarm would work (statistical independence of the two "risk factors") did not diminish the fundamental reality of the interaction between these two causal factors. In simplest terms, leaving the gas on and the fire alarm failing were independent phenomena, and yet they clearly interacted to cause a house fire.

We can summarize the epidemiologic lessons from the absent-minded Mr. Smith, in two points: (a) a pathogenic process can reach a stage at which even an unlikely exposure (with low prevalence) becomes extremely powerful in triggering the final transformation, if the subject has already undergone most of the required stages of predisposition; and (b) *a priori *knowledge of susceptibility can strongly modify our predictive ability. An important implication of the latter point is that ignorance of the genetic components of a necessary cause leads to lower estimates of the magnitude of the risk from an environmental cause (and *vice versa*). Epidemiologists are well aware that poor characterization of an exposure often leads to underestimation of its risk – our point is that poor characterization of entirely distinct components of the same sufficient cause will also lead to underestimation of a risk or even failure to detect the risk entirely.

### Genetic and acquired susceptibility

The epidemiologic search for gene-environment interactions, or (a related concept) genetic susceptibility [21], has led to some important findings, but there have been as yet few examples of dramatic differences in genetic susceptibility to environmental agents. Instead, the number of reports of modest impacts on environmentally-induced disease is steadily growing [22]. Acquired susceptibility has not received the same degree of attention as hereditary susceptibility, but increasing evidence suggests that, at least in carcinogenesis, the former (e.g. mutations relevant to carcinogenesis) is common even at birth and in the first years of life. Acquired susceptibility adds further complexity to the idea of gene environment interaction, thus making it all the more important for epidemiologists to carefully interpret the causal chains under study.

Before highlighting some recent studies on acquired susceptibility to cancer, it is important to be clear about the distinction between two similar-sounding concepts: heritability and genetic causation (or determinism). Heritability has to do with similar patterns of observable traits between parents and offspring, while a characteristic is "genetically-determined," if it is coded in and caused by the genes in a normal environment. Two extreme examples may help to clarify the distinction. The number of fingers on the human hand is completely determined by genetics – the rare deviations from 5 fingers on each hand being caused by defects of development (e.g., from thalidomide and therefore are not heritable). In contrast, wearing skirts among European populations has a very strong heritability (it occurs only in women, with the exception of the odd Scotsman). Skirt wearing is thus closely related to having two X chromosomes, but it is not genetically determined (23). Such misconceptions are clearly relevant to the discussion about the heritability versus genetic determination of cancer. For example, the study of disease clustering in identical twins does not provide clear evidence with which to infer that cancer (or schizophrenia for that matter) is due to inherited changes in DNA. Identical twins often inherit similar environments from their parents. The same applies to claims that IQ has 60% heritability, academic performance 50%, and occupational status 40%. These figures do not mean that such characteristics are inherited through genes (DNA) (i.e., that there is genetic determination, but only that there is a strong association between the characteristic in children and their parents, or between dizygotic twins as compared to monozygotic twins, depending on the study design).

#### Acquired susceptibility to cancer

One approach to studying gene-environment interactions evaluates cancer risk from exposures to carcinogens in people who have mutations shown experimentally to play a role in carcinogenesis *in vitro *or in an animal model. If such a mutation is environmentally induced, and if it increases cancer risk in humans, then this would represent a type of genetic susceptibility not from a fixed trait, but rather acquired from an environmental exposure.

Mutations can arise very early in life. A striking recent observation was the finding of a very high proportion of healthy newborns with fusion genes TEL-AML1 and AML-ETO, which are associated with lymphocytic leukaemia [24]. The frequency of these mutations in healthy newborns was about 100 times higher than the expected incidence of lymphocytic leukaemia, thereby possibly implying that this mutation, detected at birth, may be an early step in a causal chain leading to the disease. While the origin of such mutations is not known – but could reflect *in utero *exposure to genotoxicants – it is clear that these mutations alone are insufficient enough to explain the onset of leukemia, which probably requires further "hits" to the precursor cells. In agreement with this finding, Finette et al [25] found a high prevalence of *hprt *mutations at birth in healthy children.

Specific mutations caused by environmental chemicals, even early in life, may or may not constitute acquired susceptibility, but they provide evidence that such effects may be identified in the future. In a series of well-designed experiments, Somers et al [26] reported increased mutation rates in herring gulls and mice exposed to air pollution at levels that characterize normal urban environments. In mice, in fact, mutations were transmitted transgenerationally (i.e. they were attributed to DNA damage in sperm cells). Somatic mutations in newborns have been related to air pollutants [27], and mutations in germ cells have been attributed to air pollution or cigarette smoking [28,29]. In human mother-newborn pairs exposed to high levels of indoor pollution from coal smoke [30], DNA adducts and other markers reached levels higher in the newborns than in the mothers, although tranplacental exposure levels were one-tenth of the maternal exposures. In one experiment, where pregnant rats were exposed to second-hand tobacco smoke, 8-OH-dG adducts (indicating DNA damage) were formed in the fetal kidney, liver, and brain, with dose-related increases. The distribution in different organs depended on gestational stage [31]. In summary, these findings suggest that mutations can be already present at birth, predisposing to cancer if further hits occur.

#### Age effects on risk

Newborns may be particularly susceptible to the effects of carcinogen exposure. Thus, the U.S. Environmental Protection Agency analyzed animal cancer bioassay data over different periods of life [32]. Results indicated a 5- to 60-fold increased carcinogenic sensitivity in the birth-weaning period per unit dose (defined as mass/body weight^0.75 ^– day) for mutagenic carcinogens and a somewhat smaller increase – centered about 5-fold – for radiation carcinogenesis, according to Gray. The authors found a similar increased sensitivity in the fetal period for direct-acting nitrosoureas, but no such increase was detected for carcinogens requiring metabolic activation. Radiation experiments indicated that carcinogenic sensitivity is not constant through the "adult" period, but the dosage delivered in 12- to 21-month-old animals appears a few-fold less effective than the comparable dosage delivered in young adults (90–105 days of age).

The example of age is somewhat different from that of acquired susceptibility in subgroups, but it is relevant, because early exposure can be a mechanism by which highly susceptible groups arise in the population. Again, by leaving out this consideration from careful analysis, an epidemiological study may tend to underestimate the true effect of the exposure.

## Discussion

We have explored different approaches to the elucidation of causality in the epidemiology of disease arising from genes, environments, and the interplay between both. Several challenges face epidemiologists as they try to disentangle the contributions of multiple risk factors in chronic disease. Interaction is a fundamental characteristic of any causal process involving a series of probabilistic steps, thereby making it very difficult to estimate the individual contribution of any single factor in a causal chain. Routine statistical analyses are of limited help in this regard, because the standard assumptions are difficult, if not impossible, to verify. In addition, interaction is not a second-order phenomenon identified after first accounting for "main effects". Standard approaches to assessing interaction do not adequately consider the life course and the temporal dynamics through which an individual's sufficient cause is completed. Thus, different individuals may not be at the same stage of development along the path to disease, although verification is usually not possible. Further, a distinction must be made between individual-based and population-level models. Most epidemiologic discussions of causality fail to make this distinction. Finally, in quantifying interaction and assigning etiologic fractions to different necessary causes at the population level, additional uncertainty occurs because of ignorance about the components of the sufficient cause.

## Conclusion

We conclude that it is vitally important for epidemiologic research to study "interactions" and, in particular, acquired susceptibility to disease through the use of appropriate models of causation. While epidemiologists should continue to search for gene-environment interactions in the causation of chronic diseases, new insight will happen only slowly. Ignorance about steps in a causal chain will hamper the identification of component causes, whether environmental or genetic, in that chain. We therefore recommend that epidemiologists (a) pay more attention to those exposures that can induce acquired susceptibility to disease, (b) consider more thoroughly the importance of multiple exposures and their sequence in the determination of chronic diseases, and (c) appreciate that interaction is not only a statistical concept, but is deeply rooted in our models of biological causation.

## Abbreviations

ETS = environmental Tobacco Smoke

CRP = C-reactive protein

## Competing interests

The author(s) declare that they have no competing interests.

## Authors' contributions

Both authors contributed equally to this work.
